# N-Linked Glycosylation on Anthrax Toxin Receptor 1 Is Essential for Seneca Valley Virus Infection

**DOI:** 10.3390/v13050769

**Published:** 2021-04-28

**Authors:** Nadishka Jayawardena, Linde A. Miles, Laura N. Burga, Charles Rudin, Matthias Wolf, John T. Poirier, Mihnea Bostina

**Affiliations:** 1 Department of Microbiology and Immunology, University of Otago, Dunedin 9016, New Zealand; gimshan.jayawardena@oist.jp (N.J.); laura.burga@otago.ac.nz (L.N.B.); 2 Molecular Cryo-Electron Microscopy Unit, Okinawa Institute of Science and Technology Graduate University, Onna-son, Okinawa 904-0495, Japan; 3 Human Oncology and Pathogenesis Program, Memorial Sloan Kettering Cancer Center, New York, NY 10065, USA; milesl@mskcc.org; 4 Druckenmiller Center for Lung Cancer Research and Department of Medicine, Thoracic Oncology Service, Memorial Sloan Kettering Cancer Center, New York, NY 10065, USA; rudinc@mskcc.org; 5 Institute of Biological Chemistry, Academia Sinica, Taipei 115, Taiwan; 6 Laura and Isaac Perlmutter Cancer Center, New York University Langone Health, New York, NY 10016, USA; 7 Otago Micro and Nano Imaging Centre, University of Otago, Dunedin 9016, New Zealand

**Keywords:** virus receptor interaction, receptor glycosylation, picornavirus, icosahedral capsid, cryo-electron microscopy

## Abstract

Seneca Valley virus (SVV) is a picornavirus with potency in selectively infecting and lysing cancerous cells. The cellular receptor for SVV mediating the selective tropism for tumors is anthrax toxin receptor 1 (ANTXR1), a type I transmembrane protein expressed in tumors. Similar to other mammalian receptors, ANTXR1 has been shown to harbor N-linked glycosylation sites in its extracellular vWA domain. However, the exact role of ANTXR1 glycosylation on SVV attachment and cellular entry was unknown. Here we show that N-linked glycosylation in the ANTXR1 vWA domain is necessary for SVV attachment and entry. In our study, tandem mass spectrometry analysis of recombinant ANTXR1-Fc revealed the presence of complex glycans at N166, N184 in the vWA domain, and N81 in the Fc domain. Symmetry-expanded cryo-EM reconstruction of SVV-ANTXR1-Fc further validated the presence of N166 and N184 in the vWA domain. Cell blocking, co-immunoprecipitation, and plaque formation assays confirmed that deglycosylation of ANTXR1 prevents SVV attachment and subsequent entry. Overall, our results identified N-glycosylation in ANTXR1 as a necessary post-translational modification for establishing stable interactions with SVV. We anticipate our findings will aid in selecting patients for future cancer therapeutics, where screening for both ANTXR1 and its glycosylation could lead to an improved outcome from SVV therapy.

## 1. Introduction

Seneca Valley virus (SVV) is a small, non-enveloped RNA virus belonging to the genus *Senecavirus* in the family *Picornaviridae* [1]. The 7280 nucleotide (nt) long positive-sense single-stranded RNA genome of SVV comprises a 5′ untranslated region (UTR, 666 nt) and an open reading frame (ORF, 6543 nt) followed by a shorter 3′ UTR of 71 nt and a poly adenosine (poly(A)) tail of unknown length. The SVV ORF encodes a single 2181 amino acid long polyprotein, which is later cleaved into a characteristic picornaviral protein layout, L-4-3-4 [1]. In this layout, L stands for the leader protein and 4-3-4 represents the four structural proteins VP4, VP2, VP3, and VP1 and seven non-structural proteins 2A–2C and 3A–3D. In the SVV life cycle, two forms of particles exist: mature capsid with a packaged genome, and native empty capsid without RNA [2]. In both capsids, VP1 is arranged around the 5-fold axis, while VP2 and VP3 alternate around the 3-fold axis. VP4 is located in the capsid interior, forming contacts with the RNA near the 5-fold axis [2,3].

Since its serendipitous discovery, phylogenetic analyses of SVV over a span of two decades have shed light on the existence of three distinct clades [4]. SVV-001 is grouped as the lone member in clade I without any reported association to human or animal pathogenicity. SVV-001 selectively infects and replicates in tumor cells of neuro-endocrine origin and has shown promise in cancer therapeutics [5]. SVV-001 has been tested as a therapeutic agent in pre-clinical studies and in Phase I/II clinical trials for treating pediatric solid tumors and small-cell lung cancer [6,7,8,9,10]. In contrast, strains in clades II and III are linked to idiopathic vesicular disease (IVD) and epidemic transient neonatal losses (ETNL) in swine [11,12,13,14,15,16,17]. Indistinguishable characteristics between SVV-associated IVD and historical swine diseases raise caution against the possible event of a major outbreak in the agricultural industry. Thus, in-depth knowledge of the virus biology—in particular virus structure and host cell-receptor interactions—could provide beneficial information in further exploiting this virus as a cancer therapeutic or as a virus-like particle (VLP) vaccine.

To date, more than 20 cellular receptors have been identified and characterized among the members of the *Picornaviridae* family. Picornavirus receptors mainly fall into the immunoglobulin superfamily (IgSF), the structure of which consists of a single transmembrane domain and tandem repeats of two to five Ig-like domains that form contacts with the canyon in the viral capsid [18,19,20]. In addition, non-IgSF receptors, heparan sulfate [21,22], sialic acid [23], and complement control proteins act as receptors for some picornaviruses [24,25]. The high-affinity cellular receptor responsible for SVV attachment is anthrax toxin receptor 1 (ANTXR1) [26], a type I transmembrane protein with three domains: an extracellular N-terminal von Willebrand factor A (vWA) domain, a transmembrane domain, and a C-terminal cytoplasmic domain [27]. ANTXR1, also known as tumor endothelial marker 8 (TEM8), is expressed in approximately 60% of tumors but not in non-malignant cells [26]. Its homologous receptor, ANTXR2, is expressed on both healthy and tumor cells and acts as the main cellular receptor for the protective antigen of anthrax toxin secreted by the gram-positive bacterium *Bacillus anthracis* [28]. The cryo-EM structure of SVV-ANTXR1 showed that despite a large degree of structural and sequence conservation between the two receptors, the sequence along the SVV binding footprint shows divergence, thereby limiting SVV attachment only to ANTXR1 [29].

Picornavirus receptors have commonly been found to undergo post-translational modifications, specifically glycosylation [18]. This glycosylation can be grouped broadly into receptors with either N-linked glycosylation, O-linked glycosylation, or both N- and O-linked glycosylation [30]. N-glycosylation is the most prevalent type, accounting for an average of 0.94 glycosylation sites per 100 aa in mammalian receptors [31]. N-glycosylation has been well-documented for poliovirus receptor CD155 [32], coxsackievirus-A-virus (CVA) receptors ICAM-1 [33] and KREMEN-1 [34], coxsackie-B-virus (CVB) receptor CAR [35], enterovirus 71 (EV71) receptor SCARB [36], and foot-and-mouth disease virus (FMDV) receptor αVβ6 [37]. Glycan moieties were speculated to be involved in integrin receptor interactions with FMDV surface-exposed loops [37], whereas sialylation of SCARB enhanced the interactions with EV71 [36]. On the contrary, CD155 N-glycosylation in the D1 domain was shown to decrease its binding affinity to poliovirus [38]. Similar to other picornavirus receptors, the vWA domain in ANTXR1 harbors multiple glycans, which play a role in proper folding and trafficking of the receptor to the cell surface [39]. However, the functional role of ANTXR1 glycosylation with respect to SVV attachment or entry remains unknown.

In this study, we characterized the glycosylation of recombinant ANTXR1 extracellular domain fused to a fragment-crystallizable (Fc) region and its importance in SVV-ANTXR1 interactions and cellular infection. Glycopeptide analysis of the recombinant ANTXR1 recognized three N-linked glycosylation sites in total: two localized to the ANTXR1 extracellular domain and one located in the Fc region. Local cryo-EM map refinement of the SVV-ANTXR1 asymmetric unit further confirmed the presence of N-glycosylated sites at N166 and N184 in the ANTXR1 vWA domain. A combination of co-immunoprecipitation assays and cellular infection experiments showed the necessity of ANTXR1 glycosylation for successful SVV attachment and cellular entry. The findings from this study could provide valuable insights into selecting cancer patients for SVV treatments or in designing inhibitors that can specifically block N-linked glycans in ANTXR1 to prevent SVV infection in swine.

## 2. Materials and Methods

### 2.1. SVV Purification

SVV purification was carried out according to previously published protocols [2]. Briefly, 8 T175 cm^2^ flasks of H446 wild-type (wt) cells (ATCC, HTB-171) were grown in Roswell Park Memorial Institute (RPMI) 1640 medium (Catalog no. 1851354, Gibco, Waltham, MA, USA) and 10% fetal bovine serum (FBS). At ~80% confluency, the medium was replaced with RPMI-1640 plus 2% FBS, and cells were infected with plaque-purified SVV at a multiplication of infection of 1, followed by incubation at 37 °C for 48 h. Once the cytopathic effect (CPE) was confirmed after 48 h, cells were subjected to 3 repeated cycles of freeze-thawing to lyse the cells and release the virus. Lysed cells and medium were subjected to high-speed centrifugation at 10,000× *g* for 30 min at 22 °C to pellet down the cells. The resulting supernatant was centrifuged at 120,000× *g* for 1 h at 4 °C using a Beckman Coulter SW32Ti rotor. The virus pellet was resuspended in CsCl purification buffer (137 mM NaCl, 5 mM KCl, 25 mM Tris base, 0.8 mM NaH_2_PO_4_) at 4 °C, overnight. Resuspended virus was loaded onto a 1.33 g/mL CsCl isopycnic gradient and centrifuged at 61,580× *g* for 18 h at 22 °C. The band corresponding to SVV full capsids was collected using an 18-gauge needle and dialyzed overnight in PBS at 4 °C.

### 2.2. SVV-ANTXR1 Interaction

The concentration of purified SVV capsids was measured by using a Qubit^TM^ protein assay kit according to the manufacturer’s instructions [40]. The interaction between SVV and ANTXR1-Fc was carried out according to methods adapted from previously published protocols [29]. Purified SVV stock (0.2 mg/mL) and recombinant human ANTXR1-Fc (1 mg/mL) (NP_115584.1; Met1-Ser321, Catalog no. 13367-H02H, Sino Biological, Beijing, China) were mixed in equal volumes and incubated at 37 °C for 1.5 h, followed by incubation at 4 °C for 1.5 h. Recombinant ANTXR1-Fc was expressed and purified from HEK293 cells.

### 2.3. Glycopeptide Analysis of ANTXR1 Glycosylation Sites

Glycopeptide analysis was performed by the University of Georgia Complex Carbohydrate Research Center. Recombinant human ANTXR1-Fc was subjected to acetone precipitation to remove excess Tween 80 in the preparation. Precipitated proteins were then reduced and alkylated, followed by dialysis in Milli-Q water. Dialyzed sample was dried and ~120 µg of aliquot was measured and divided into 3 aliquots of 40 µg each. Three aliquots were digested with trypsin, chymotrypsin, and Glu-C. A portion of each digest was deglycosylated with PNGase F in ^18^O Water (H_2_^18^O). Each digest was then profiled by LC-nSI-MS/MS (Orbitrap-Fusion with EASY nanospray source and Ultimate 3000). The LC separation was performed on a nano-C18 column using water/acetonitrile gradient with formic acid. MS/MS was acquired with 3 different fragmentation methods (collision induced dissociation (CID), electron transfer dissociation (ETD), and higher-energy collisional dissociation (HCD)) for each peptide/glycopeptide ion during the LC-MS run. The resulting spectra for each run were analyzed manually by searching for the targeted glycopeptide ions. The structural assignments were annotated based on the observed ion mass as well as the MS/MS fragmentation data.

### 2.4. Co-Immunoprecipitation Studies

SVV was cultured, purified, and titered as previously described [9,41]. For co-immunoprecipitation experiments, recombinant ANTXR1-Fc protein (0.25 µg) was incubated with magnetic Protein G Dynabeads (Invitrogen, Carlsbad, CA, USA; 30 mg/mL; 1 µL) for 10 min at room temperature in PBS, pH 7.4. Bead-Fc complexes were then incubated in the presence or absence of PNGase F (500 units; NEB, Ipswich, MA, USA) in 1× Glycobuffer (NEB) for 0–24 h at 37 °C. Complexes were subsequently washed and incubated with SVV-001 (2.0 × 10^10^ vp) for 2 h at 4 °C. Triplicate washes of the complexes were performed with PBS pH 7.4 supplemented with 0.02% Tween-20 (Sigma, St. Louis, MO, USA), and the bead-Fc-protein complexes were eluted by boiling for 10 min at 90 °C in RIPA buffer supplemented with NuPAGE sample reducing agent and LDS sample buffer (Invitrogen, Carlsbad, CA, USA). Western blotting was performed as described previously [26]. Duplicate protein samples were resolved on a 4–12% Bis-Tris polyacrylamide gel with MOPS running buffer (Invitrogen, Carlsbad, CA, USA). The gel was then stained using the Pierce Silver Stain kit and imaged using a E-Gel Imager (Invitrogen, Carlsbad, CA, USA).

### 2.5. Cell Blocking Studies

SVV-GFP reporter virus contains a fusion protein of GFP flanked with 2A sequences and was cultured, purified, and titered as previously described [9,41]. SVV-GFP infectious titers were determined as described previously by flow cytometry [42]. H446wt cells were seeded in a 6-well tissue culture treated plate (Corning, Corning, NY, USA) 16–24 h prior to infection and incubated overnight at 37 °C. ANTXR1-Fc protein (5 µg/mL) was incubated in the presence or absence of PNGase F (2.5 × 10^3^ units) in 1× Glycobuffer for 24 h at 37 °C and subsequently incubated with SVV-GFP (MOI = 5) in PBS for 1 h on ice. Protein-virus solution was then added to cells for 16 h at 37 °C. Cells were then incubated with NucBlue Live ReadyProbe reagent (Invitrogen, Carlsbad, CA, USA) for 20 min at 37 °C. Cell images were obtained with an EVOS FL Auto fluorescence microscope (Invitrogen, Carlsbad, CA, USA).

### 2.6. Plaque Formation Assay

H446wt cells were seeded in a 12-well plate at 70% confluency. Next day, the confluent monolayers of H446wt cells were infected with serial dilutions of SVV in RPMI1640. After 1 h of incubation at 37 °C, virus dilutions were removed from the plates and each well was immediately overlaid with RPMI1640 supplemented with 2% FBS and 1% agarose (SeaPlaque; catalog number 50100; Lonza, Basel, Switzerland). The plates were stained with 5 mg/mL 3-(4,5-dimethylthiazol-2-yl)-2,5-diphenyltetrazolium bromide (MTT; catalog number M2128; Sigma, St. Louis, MO, USA) for plaque visualization after 24 h incubation at 37 °C. For deglycosylation assay, H446wt cells were pre-treated with 100 units of PNGase F for 1 h at 37 °C and the excess liquid was removed prior to infection with SVV.

### 2.7. Focused Classification/Refinement of SVV-ANTXR1 Asymmetric Unit and Modeling of Glycans

The protocols related to cryo-EM data used in this study were previously published in our study describing the SVV-ANTXR1 structure [29]. To better visualize the glycosylation sites in ANTXR1-Fc bound to SVV we sought to perform a localized reconstruction of one asymmetric unit. To achieve this, imposed I4 symmetry in the particle stack from final 3D refinement was relaxed to C1 symmetry using the *relion_particle_symmetry_expand* command, thus resulting in a particle stack of 406,920 symmetry related copies. This particle stack was subjected to 3D classification in Relion-3.1 using a custom mask created by UCSF Chimera “Volume eraser” tool, to include one asymmetric unit in SVV-ANTXR1-Fc, masking out the remainder of the complex. A cryo-EM map of SVV-ANTXR1-Fc low-pass filtered at 30 Å resolution was used as the starting reference. 3D classes from the latter step were further subjected to masked 3D refinement and post-processing in Relion-3.1 using the same 3D mask as before. Spatial resolution of the final reconstruction was calculated to be 6.6 Å based on Fourier shell correlation cut-off of 0.143 between two independent half-maps.

To model the glycosylation site on ANTXR1-Fc, our previously published cryo-EM structure of SVV-ANTXR1 (PDB ID 6XC1) was first manually fitted into the cryo-EM map using UCSF Chimera [43]. Glycan tree accounting for the additional density in the cryo-EM map was manually built using the carbohydrate module in Coot [44]. All the figures were generated in UCSF ChimeraX.

## 3. Results

### 3.1. Profiling of Glycosylation Sites in ANTXR1

As a first step toward recognizing potential N-linked glysosylation sites in ANTXR1-Fc recombinant preparations used in this study, we combined different proteolytic degradation protocols followed by deglycosylation with PNGase F in the presence of ^18^O water to obtain glycopeptides suited for LC-nSI-MS/MS analysis. ANTXR1-Fc from Sino Biological was expressed and purified from HEK293 cells and therefore, was subject to similar glycosylation modifications as endogenous ANTXR1. PNGase F acts as a broad-spectrum enzyme that cleaves all N-linked hybrid or complex oligosaccharides found in plants and mammals, except in the presence of a core α1, 3 fucose connected to asparagine-linked N-acetylglucosamine (NAG) [45,46]. In such instances, the prior denaturation of protein is essential for an efficient cleavage by PNGase F. Therefore, we treated separate aliquots of ANTXR1-Fc with trypsin, chymotrypsin, and Glu-C. A combination of different proteases allowed us to obtain spectra of glycopeptide ions accounting for all potential N-glycosylation sites. A portion of each digest was deglycosylated with PNGase F in ^18^O water to convert the glycan-modified asparagine to an ^18^O aspartic acid residue, causing a subtle mass shift of 3 Da [47].

Our ^18^O labeling experiment recognized three N-linked sites at residues N166 and N184 in the extracellular vWA domain of ANTXR1 and at N81 in the fused Fc domain (Table 1). These findings were in agreement with previously discovered N-linked glycosylation sites in both ANTXR1 and ANTXR2 [39]. In our analysis, N166 and N184 were found to be heavily glycosylated. All N166 sites showed 100% glycan occupancy, while N184 sites were mostly glycosylated (Table 1). These glycosylated sites in the vWA domain carried both bi-antennary and tri-antennary sialylated and fucosylated complex glycans. Notably, some of the glycans in latter N-linked sites contained a LacdiNAc antenna in the structure. In addition, one sulfated N-glycan was shown to be linked to N184. Overall, the high degree of LacdiNAc expression, heavy fucosylation, and sulfation observed here showed similarities to glycoprotein expressed in human cell lines [48].

### 3.2. Cryo-Electron Microscopy Reconstruction of ANTXR1 Glycosylation Sites

**Figure 1:**
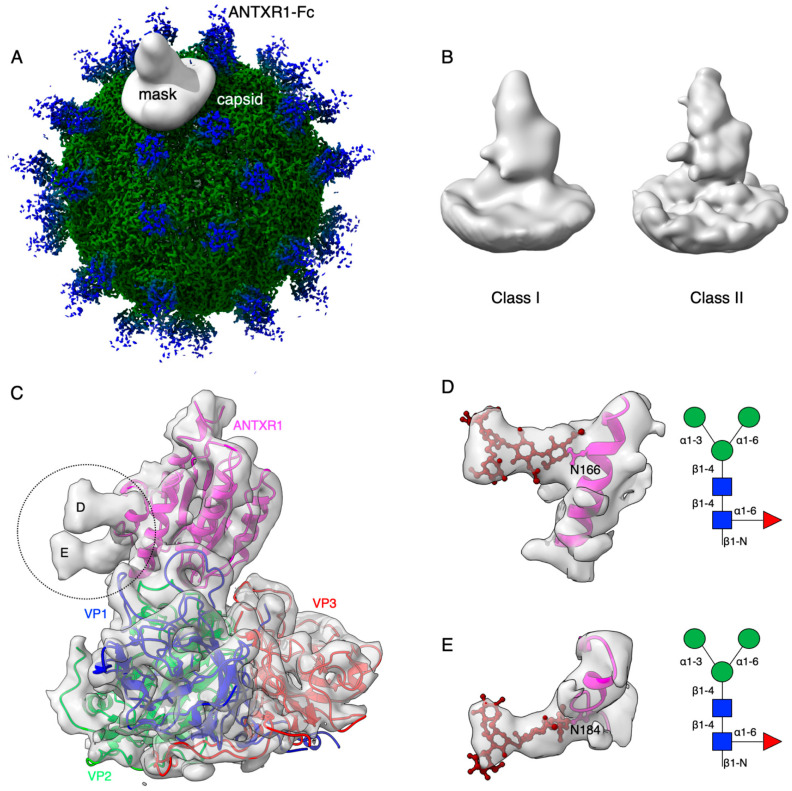
Symmetry-expanded cryo-EM reconstruction of SVV-ANTXR1-Fc asymmetric unit. (**A**) Cryo-EM reconstruction (map contoured at 3.66 σ above average) of SVV-ANTXR1-Fc complex at 3.8 Å resolution (EMD-7772). A soft-edged mask enclosing one asymmetric unit was applied to symmetry-related copies of the particle. (**B**) All four 3D classes generated from classification of masked symmetry-expanded particles display features of receptor glycosylation. (**C**) Electron potential map (map contoured at 0.34 σ above average) corresponding to one SVV capsid asymmetric in complex with ANTXR1 vWA domain. Blue, green, red and magenta colored ribbons represent VP1, VP2, VP3, and ANTXR1, respectively. Masked-out cryo-EM densities for N166 (**D**) and N184 (**E**) glycosylation sites are located on the α4 helix and α5-β5 loop of ANTXR1. Fitted atomic glycan coordinates modeled as the common glycan composition are shown in maroon ball-and-stick representation. Common glycan composition from N-link to the first branching site of the latter two sites is shown in the schematic diagrams (**D**,**E** right, and red-dashed box in Table 1). Fucose, N-acetylglucosamine and mannose are depicted by red triangles, blue rectangles, and green spheres, respectively.

To further visualize and dissect the role of ANTXR1 glycosylation in the context of interactions with SVV, we reprocessed the data from our previously published SVV-ANTXR1 cryo-EM structure (Figure 1A). In the original cryo-EM reconstruction, imposing strict icosahedral symmetry averaging during the reconstruction process attenuated the signal of asymmetric or structurally heterogeneous features such as glycans due to their local variability. In order to overcome this issue, we used local symmetry expansion, followed by an established focused classification and orientation refinement approach to resolve these local glycan features more clearly. In this method, the particle stack from the final 3D refinement of SVV-ANTXR1 was symmetry-expanded [49], thus creating a stack containing symmetry related copies [50]. Then, we applied a soft-edged mask to cover one asymmetric unit of the reconstruction plus a distance of 12 pixels extended in all directions (Figure 1A). Initial 3D classification of the data set did not recognize any distinct structural heterogeneity and had similar features (Figure 1B), which led us to refine the entire data set to obtain the final cryo-EM map of the SVV-ANTXR1 asymmetric unit at 6.6 Å resolution (Figure 1C).

In agreement with our glycopeptide profiling, we observed two glycosylation sites at N166 and N184 in the extracellular vWA domain of ANTXR1 (Figure 1D,E). We could observe and fit the coordinates corresponding to a complex glycan profile, starting from the N-link up to its first bisection, a composition common to all of the glycans detected by LC-nSI-MS/MS analysis (red-dashed box, Table 1). Features in the cryo-EM electron potential map corresponding to N166 appeared to be better resolved compared to map detail at the N184 site as evident from the appearance of glycan branching at a lower threshold (contour level of 4 σ vs 3.4 σ above average). This suggested a higher degree of order within the glycans at N166 in comparison to N184, as shown by the MS analysis. In picornavirus receptors, N-linked glycans are not involved in direct interactions with the capsid, except in the case of interactions between αVβ6 and FMDV, where N266 linked glycan was shown to extend to the capsid surface, presumably bridging the integrin to the virus surface [37]. ANTXR1 vWA adopts a similar “Rossman fold” to that of αVβ6 and interacts with the surface-exposed loops in VP1-VP3, reminiscent of FMDV- αVβ6 interactions. However, both N-linked glycans in the ANTXR1 vWA domain extend parallel to the capsid floor. Thus, neither N-linked glycan at N166 nor N184 was in close proximity to SVV surface-exposed loops, excluding a direct role of these glycans in mediating virus contact.

### 3.3. ANTXR1 Glycosylation Is Essential for SVV Attachment and Entry

**Figure 2:**
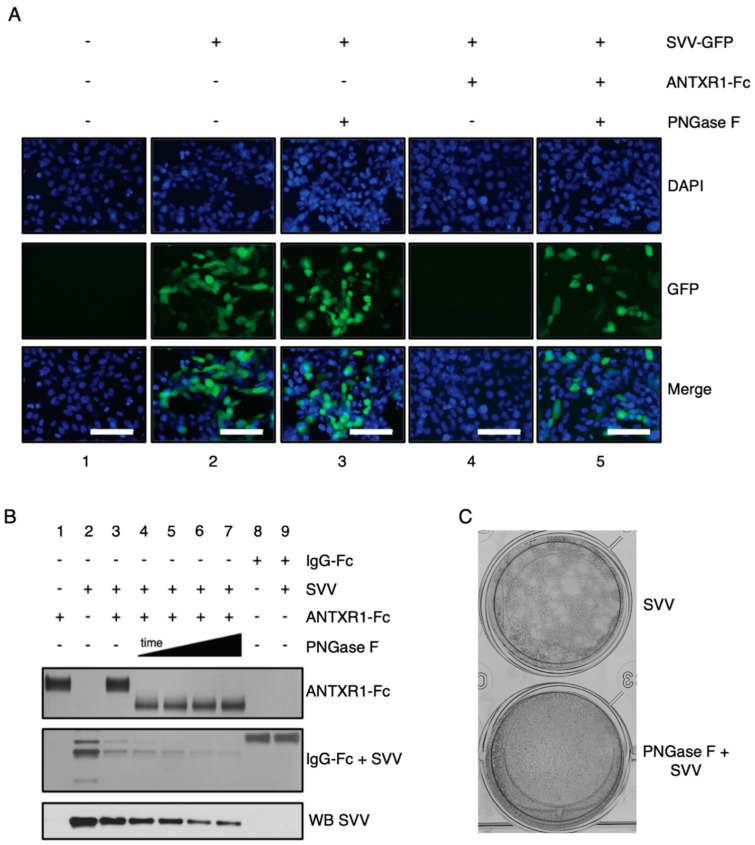
ANTXR1 glycosylation is a critical step for SVV attachment and entry into H446 cells. (**A**) Fluorescence microscopy images depicting infection of H446wt cells with SVV-GFP. ANTXR1-Fc was incubated in the absence (lane 4) or presence (lane 5) of PNGase F, followed by incubation with SVV-GFP and subsequent infection of cells. Uninfected cells (lane 1), cells infected with SVV-GFP alone (lane 2), or SVV-GFP incubated with PNGase F were included as controls. Scale bar, 200 µm. (**B**) Co-immunoprecipitation experiments of ANTXR1-Fc and SVV. Silver-stained SDS-PAGE depicting ANTXR1-Fc (top panel) and SVV capsid /IgG-Fc proteins (middle panel) and corresponding immunoblot (bottom panel) of co-immunoprecipitation. SVV was incubated with ANTXR1-Fc (lanes 3–7) complexed to Protein G Dynabeads. ANTXR1-Fc was pretreated with PNGase F for increasing periods of time (lanes 4–7; 4 h, 8 h, 16 h, 24 h) prior to incubation with SVV. ANTXR1-Fc alone (lane 1), SVV input (lane 2), IgG-Fc alone (lane 8), or IgG-Fc after incubation with SVV (lane 9) are shown as controls. (**C**) Plaque formation assay of SVV incubated with H446wt cells (left) or H446wt cells pre-treated with PNGase F (right).

In order to assess if N-glycosylation on ANTXR1 is necessary for successful viral attachment and entry, we performed infection studies with GFP-tagged virus (SVV-GFP) and ANTXR1-Fc protein that was pre-incubated in the presence or absence of PNGase F (Figure 2A). Visualization of SVV-GFP infected H446wt cells by fluorescence microscopy revealed that when SVV-GFP is pre-incubated with ANTXR1-Fc, all of the receptor binding sites on the SVV capsid become saturated, ergo blocking viral entry into cells. However, in the presence of PNGase F-treated ANTXR1-Fc, SVV is internalized into H446wt cells, clearly demonstrating loss of interaction with deglycosylated ANTXR1-Fc. In two control groups, we either treated H446wt cells with SVV-GFP alone or SVV-GFP incubated with PNGase F. Virus internalization was visible in both control groups and eliminates issues related to virus infectivity or H446wt receptor deglycosylation by partially inactivated PNGase F. We also performed co-immunoprecipation assays to examine the effect of PNGase treatment of ANTXR1-Fc (Figure 2B). We found that exposure of ANTXR1-Fc with PNGase F for 4 h induced a significant change in the molecular weight of ANTXR1-Fc due to deglycosylation. Additionally, we observed a time-dependent decrease in the amount of SVV protein bound to the PNGase F-treated ANTXR1-Fc. Alternatively, H446wt cells were directly incubated with PNGase F for 1 h at 37 °C prior to infection with 10^−5^ dilution of the SVV stock. Plaque formation assays (PFA) confirmed no visual plaques on PNGase F-treated cells compared to non-treated cells (Figure 2C). Even more, cryo-EM experiments aiming to observe SVV capsids decorated with deglycosylated ANTXR1 did not show any density corresponding to receptors (data not shown), suggesting that ANTXR1 adopts a different conformation incapable of binding with the SVV capsid. Collectively, these results show that ANTXR1 glycosylation is important for successful SVV attachment and entry.

## 4. Discussion

Glycosylation in viral proteins or their receptors is a highly regulated posttranslational modification affecting their tertiary structure, function, stability, and downstream cellular signaling pathways. Viral protein or receptor glycosylation can generally be broadly categorized into two main groups: N-linked and O-linked. In N-linked glycosylation, glycans are attached to the asparagine residue in an Asp-X-Ser/Thr sequon, where X can be any amino acid except for proline. In O-linked glycosylation, the sugar moiety is attached to the oxygen atom of a serine or threonine residue. Viral protein glycosylation has been reported for enveloped viruses such as coronavirus spike protein [51], ebola virus glycoprotein [52], hemagglutinin glycoprotein of influenza virus [53], envelope glycoproteins of flaviviruses [54,55], and Lassa virus envelope glycoprotein [56]. Although multiple roles have been suggested for glycosylation with respect to viral pathogenesis, immune evasion by heavy glycan shielding or using shed/secreted glycoproteins remains the primary role [57]. Second to this role, glycans in viral envelope proteins act as attachment factors [57,58]. Alternatively, for non-enveloped viruses such as adenoviruses [59], rotaviruses [60], reoviruses [61], and picornaviruses [23,62], this mechanism reverses to utilize cell surface sialic acid/poly-sialic acid or glycans on cellular receptors as attachment factors.

**Figure 3:**
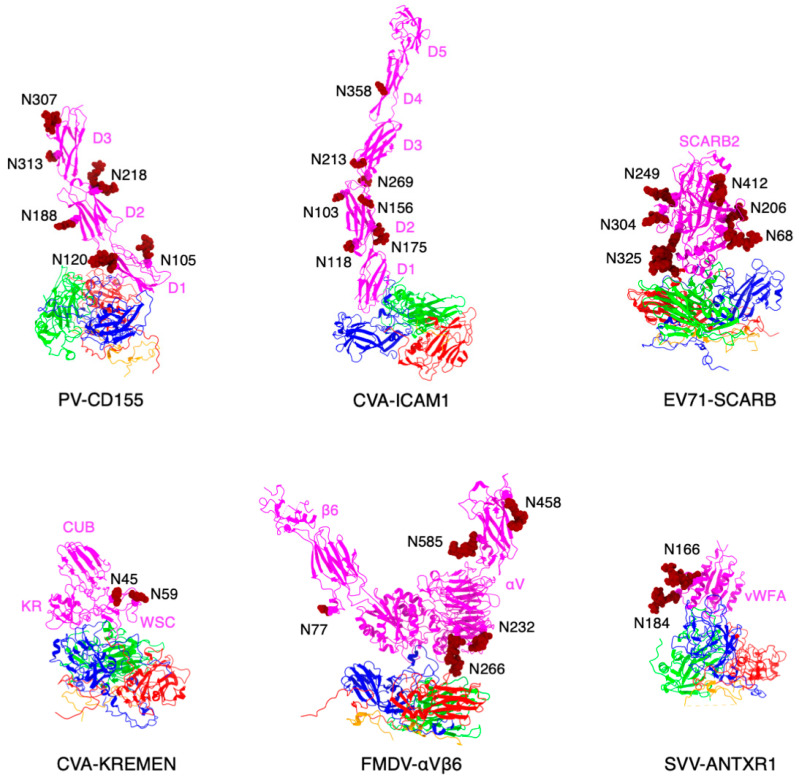
Glycosylation of picornavirus receptors. Poliovirus (type I Mahoney strain) in complex with CD155 extracellular domains D1-D3 (PDB: 3J8F). Interaction of coxsackievirus A21 with the extracellular D1-D5 domains of ICAM-1 (PDB: 1Z7Z). Enterovirus 71 in complex with SCARB2 (PDB: 6I2K). Coxsackievirus A10 bound to ectodomain of KREMEN (6SNW). KREMEN ectodomain comprises three equally-sized subdomains: kringle (KR) containing transmembrane protein, cell wall stress-responsive component (WSC), and complement C1r/C1s, Uegf, Bmp1 domain (CUB). FMDV strain O1M and its cellular receptor αVβ6 integrin (5NEM). Virus receptor complex of SVV and vWFA domain of ANTXR1 (6CX1).

In picornaviruses, the usage of sialic acid as an attachment factor has been reported for members in the genus *Enterovirus* such as CVA24v, EV68, EV70, and EV71 [23,62]. Glycosylation sites observed in picornavirus receptors describe a rich geometry (Figure 3). For instance, N-linked glycans in αVβ6 integrin form additional contacts between the receptor and surface-exposed loops of FMDV capsid proteins [37]. Interestingly, heavy N-linked glycosylation in CD155 abrogates poliovirus infectivity [38], a testament to plausible steric hindrance by surrounding glycans when the receptor needs to reach the capsid floor to complete its interaction with the capsid, as opposed to forming contacts with surface-exposed loops. Hence, picornaviruses are a group of viruses in which receptor glycosylation can be expressed as a “double-edged sword”, rather than an exclusively beneficial receptor modification favoring viral entry in every case.

To further our knowledge of the functional role of receptor glycosylation in picornaviruses, we investigated the glycosylation in the SVV receptor, ANTXR1. In agreement with previous literature, our ^18^O labeling experiment in conjunction with LC-MS analysis revealed two glycosylation sites at N166 and N184 in the extracellular vWA domain of ANTXR1 and one glycosylation site at N81 located in the fused Fc domain. N-glycosylation in ANTXR1 has also been shown to be essential for protein folding, stability, trafficking, and ligand binding [39]. This pattern of N-glycosylation in ANTXR1 is also observed in ANTXR2, albeit less dependent on its functional roles. The presence of N-glycans in the vWA domain in ANTXR1 was visible in its complexed state with SVV. Symmetry expanded reconstruction of the SVV-ANTXR1 asymmetric unit confirmed the presence of two N-glycosylation sites in the vWA domain, which extended parallel to the SVV capsid surface, therein demonstrating no contacts with surfaced-exposed loops. Despite the absence of any interactions between ANTXR1 glycans and SVV capsid, ANTXR1 deglycosylation was able to abrogate viral entry into the small-cell lung cancer cell line H446wt, highlighting its stringent effect on receptor function. While the effect of N-glycosylation on the conformational stability of ANTXR1 and ANTXR2 has not been extensively investigated, it has been shown that N-glycosylation is essential for conformational stability in extended open conformation of integrins [63]. The same observation presumably holds true for ANTXR1, ligand binding open conformation, which closely resembles the integrin open state. This could explain the complete loss of interactions, subsequent viral entry, and infection when ANTXR1 is deglycosylated by PNGase F.

Our results immediately suggest two important therapeutic applications: (1) As a biomarker for screening for patients with glycosylated receptors in tumors, as we have shown that glycosylation is essential for SVV binding, entry and subsequent infection. (2) The specific role of ANTXR1 in swine remains unknown and this eliminates the possibility of using enzymatic cleavage of ANTXR1 glycans. Nonetheless, as shown with other viruses such as influenza and HIV, broad spectrum lectins can be directed against heavily fucosylated and sialylated glycans in ANTXR1 with the overarching goal of triggering a steric hindrance in the vicinity of the SVV binding site.

## Figures and Tables

**Table 1:** Glycan profiles of N166, N184, and N81 (Fc) glycosylation sites as identified by LC-nSI-MS/MS analysis.

Profiled Glycan		N166	N184	N81 (Fc)
Deoxyhex_1_HexNAc_5_Hex_4_	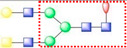	✓		✓
Deoxyhex_2_HexNAc_4_Hex_5_	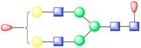	✓	✓	
Deoxyhex_2_HexNAc_5_Hex_4_	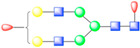	✓	✓	
Deoxyhex_1_HexNAc_5_Hex_5_	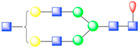	✓	✓	✓
Deoxyhex_1_HexNAc_4_NeuAc_1_Hex_5_	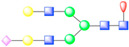	✓	✓	✓
Deoxyhex_1_HexNAc_5_NeuAc_1_Hex_4_	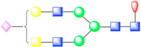	✓	✓	
Deoxyhex_1_HexNAc_5_NeuAc_1_Sulph_1_Hex_4_	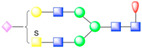		✓	
Deoxyhex2HexNAc4NeuAc1Hex_5_	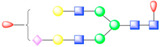		✓	
Deoxyhex_2_HexNAc_5_NeuAc_1_Hex_4_	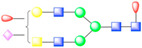		✓	
Deoxyhex_1_HexNAc_5_NeuAc_1_Hex_5_	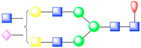	✓	✓	
Deoxyhex_1_HexNAc_4_NeuAc_2_Hex_5_	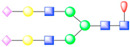	✓	✓	
Deoxyhex_1_HexNAc_5_NeuAc_2_Hex_4_	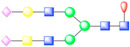		✓	
Deoxyhex_1_HexNAc_5_NeuAc_2_Hex_5_	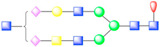		✓	
Deoxyhex_1_HexNAc_6_NeuAc_1_Hex_6_	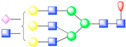		✓	
Deoxyhex_1_HexNAc_5_NeuAc_2_Hex_6_	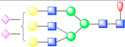	✓	✓	
Deoxyhex_1_HexNAc_6_NeuAc_2_Hex_6_	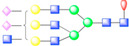	✓	✓	
Deoxyhex_1_HexNAc_5_NeuAc_3_Hex_6_	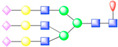		✓	

Fucose: red triangle, N-acetylglucosamine: blue rectangle, mannose: green sphere, galactose: yellow sphere, N-Acetylneuraminic acid: purple diamond. Common sugar composition for all shown glycans is enclosed within the red dashed box. The presence of different glycans at each position is shown by ✓.

## Data Availability

Not applicable.
